# Health care seeking behavior of parents with acute flaccid paralysis child

**DOI:** 10.11604/pamj.supp.2017.27.2.11023

**Published:** 2017-06-09

**Authors:** Ayesheshem Ademe Tegegne, Amare Mengistu Mersha

**Affiliations:** 1 World Health Organization Country Office, Addis Ababa, Ethiopia

**Keywords:** Health care seeking behavior, acute flaccid paralysis, disease surveillance, child

## Abstract

**Introduction:**

Despite the tremendous increase in the number of modern health institutions, traditional medical practices still remain alternative places of health care service delivery and important sites for disease notification in the disease surveillance system. The objectives of this study are to describe the patterns and factors associated with health care seeking behavior of parents and care takers with acute flaccid paralysis child and see how the traditional practice affect the surveillance system.

**Methods:**

A cross-sectional descriptive study was conducted to assess the health seeking behavior of parents with an acute flaccid paralysis child. Data were collected throughout the country as a routine surveillance program.

**Results:**

Of 1299 families analyzed, 907(69.3%) of families with AFP child first went to health institutions to seek medical care, while. 398 (30.7%) of parents took their child first to other traditional sites, including holy water sites (11.8%), traditional healers (9.1%) and prayer places (5.4%). Over half of the parents with AFP child reported practicing home measures before first seeking health service from modern health institutions. Home measures (OR, 0.1202, 95% CI 0.0804-0.1797), decision by relatives (OR, 0.5595, 95% CI 0.3665-0.8540) and More than 10km distance from health facility (OR, 0.5962, 95% CI, 0.4117-0.8634) were significantly associated to first seeking health service from health institutions (p<0.05).

**Conclusion:**

Program strategies must certainly be developed to expand and capture all traditional sites in the surveillance network, and intensify sensitization and active surveillance visit in these areas.

## Introduction

Poliomyelitis is a serious infectious disease that primarily affects young children causing permanent disabilities and deaths. In 1988, when the World Health Assembly passed a resolution to eradicate polio by the year 2000, the annual number of paralytic polio cases was estimated at 350,000 worldwide [1]. Since then, substantial progress has been made in the eradication of poliomyelitis globally, and three WHO Regions, the regions of the Americans (1994), Western Pacific Region (2000), and European Region (2002) have already been certified free of indigenous wild polio virus [2–4]. As of October 10, 2016, the numbers of polio cases have been dramatically reduced and clustered in a few geographic areas from what has been reported in 1988 [5]. However, global eradication of poliomyelitis remains a concern for all countries.

Available evidence indicates that Ethiopia suffered a lot from poliomyelitis infection. A study conducted in 1988 in Gondar zuria in the Northwest Ethiopia among children aged 1-15 years old indicated a prevalence of residual poliomyelitis of 2.1/1000, while an estimated annual incidence of poliomyelitis was found to be 7.7/100000 [6]. Another study also demonstrated a prevalence of paralytic poliomyelitis of 7.3/1000 children in 5-9 years old [7]. Ethiopia, following the Yaoundé declaration on polio eradication in 1996, joined the global effort for the eradication of poliomyelitis and established in 1997 acute flaccid paralysis case-based surveillance system throughout the country [8]. Progress has been made for the implementation of polio eradication strategies in terms of surveillance networking, capacity building, and supplemental immunization activities (SIAs). Nonetheless, the achievements were suboptimal at the beginning according to the major indicators of quality and sensitivity of the surveillance system i.e. stool adequacy within 14 days of onset of paralysis and non-polio acute flaccid paralysis (NP-AFP rate) [9]. The trend of stool adequacy and nonpolio AFP rates were persistently suboptimal for certification in spite of the regular active case search and sensitization efforts at all level. From the start of the acute flaccid paralysis surveillance up to the year 2000, the NP-AFP rate had persistently been lower than 0.8/100,000 children < 15 years old, while stool adequacy was lower than 50 % during the same period [10].

The Ethiopian health service system is primarily focused on the preventive and promotive aspect of health service. The health service system is designed on a three-tier health service delivery system, which comprises a primary health care unit, (a network of a health center and five health posts), general hospital, and specialized hospital. The primary health service coverage is estimated at 76.9%, while health service utilization is at 33% [11]. A longitudinal community based study conducted from 1992-1994 on mothers health seeking behavior for ill babies at various levels indicated that less than 47% of ill children only got treatment for various illness [12]. Hence, the objective of this study is to investigate the health care seeking behavior and related factors inhibiting families or care takers of acute flaccid paralysis to visit first modern health facilities.

## Methods

Acute flaccid paralysis cases were reported from all regions, zones, and woredas (equivalent to district) of the country. Treatment for illness is available from public, private health facilities, traditional practitioners, and holy water sites. Under the polio eradication program, all children under the age of 15 years that present with sudden onset of flaccid paralysis are investigated and reported immediately. All professionals are also required to investigate and report any suspected AFP cases without delay. As soon as cases notified arrangements are made for two stool samples collected within 24 hours apart and within 60 days but preferably within the first 14 days of onset of paralysis. Samples are transported to the National Polio laboratory at the Ethiopian Health and Nutrition Research Institute within 72 hours with appropriate cold chain system (2-8Oc). The WHO medical surveillance officer of that area is required to verify, investigate the case and fill health care seeking questionnaire. All cases investigated by medical surveillance officers from August 2004 to May 2008 throughout the country were included in the study and retrospectively analyzed.

A standardized and pretested questionnaire was used to collect information on health care seeking behavior of parents and caregivers of acute flaccid paralysis child after securing verbal consent from respondents. The questionnaire includes demographic characteristics of the parents and epidemiologic characteristics of the cases. Part of the questionnaire includes socio- demographic variables like income, religion, education, occupation, specific measures taken at home, the first site of visit, reasons for choosing the first site and awareness on AFP and polio eradication initiatives. Data were entered, summarized, and analyzed using EpiInfo 2000 version 3.2 statistical packages. Five records with incomplete information on the date of onset and first site visited were excluded from the analysis. We used simple frequencies and bivariate analysis with further analysis with multivariate logistic regression.

## Results

**Figure 1:**
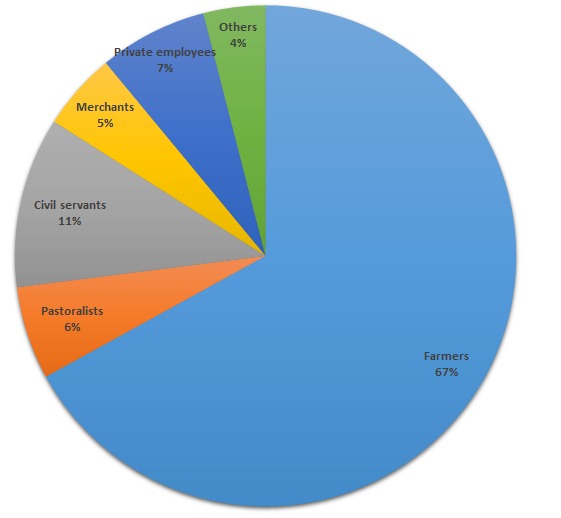
Distribution of occupations of respondents of families with acute flaccid paralysis child, Ethiopia, 2008

A total of 1338 caretakers with acute flaccid paralysis child were interviewed including examination of their children with AFP and health seeking questionnaires were filled. Of these analyzed, 797(60%) are females. Nearly half (49.7%) of the respondents were the child´s mothers, while one-third were fathers, and only 10.9% of the respondents were both parents. The majorly (70.1%) of the respondents were of the Orthodox Christian faith and two-thirds of the respondents had no schooling at all. Sixty seven and a half percent (67.5%) of the respondents were farmers and the majorities (70%) were living on an income less than 200 Ethiopian Birr (ETB) per month. Ninety percent of the respondents were from South Nation's and Nationalists Region (SNNPR), Oromia, Amhara, Tigray and Addis Ababa Regions. The median number of days from the date of onset to the detection of cases at facility level was found to be 6 days, while it ranged from less a day to 60 days (Table 1, Figure 1).

**Table 1:** Distribution of families with AFP child by selected variables 2004-2008, Ethiopia

Variables		
**Sex respondent**	**Frequency**	**Percent**
Male	509	40.0
Female	797	60.0
**Total**	1306	100
**Religion of respondents**		
Orthodox Christian	937	70.1
Muslims	385	28.8
Others	14	1.0
**Total**	1336	100
**Education of respondents**		
No formal education	839	67.6
Primary	249	20.0
Secondary	122	9.8
Tertiary	32	2.6
Total	1242	100
**Income of respondents in ETB**		
<200	854	70.0
200-400	190	15.6
401-600	86	7.0
601-800	51	4.2
>800	39	3.2
**Total**	1222	100
**Sex (child)**		
Female	573	43.3
Male	751	56.7
**Total**	1324	100
**Child age**		
<5	846	68.1
6-10	279	22.44
11-15	118	9.5
Total	1243	100
**Vaccinated in routine immunization**		
Yes	1032	85.1
No	180	14.9
Total	1212	100
**Vaccine doses child received**		
0 dose	48	4.9
1-2 doses.	127	13.1
3+ doses	762	78.6
Unknown	33	3.4
Total	957	100

The male: female ratio of AFP cases was 1.5:1. Eight hundred and forty one (68.1%) and 397(31.9%) of the AFP cases were under five years of age, and between 6-15 years of age respectively. In regard to vaccination history, 1032(85.5%) of parents indicate that their children have taken at least one dose of OPV in routine or during vaccination campaigns. Of those children immunization status evaluated, 889(91.7%) of these have taken at least one dos OPV, 762 (78.6%) of the kids were fully immunized for OPV (>=3 doses of OPV) and 127(13.1%) have taken<=2 doses OPV. On the other hand, less than five percent (4.9%) of the kids haven´t taken any dose of OPV, while 33(3.4%) of the kids have unknown histories of vaccination (Table 1).

**Figure 2:**
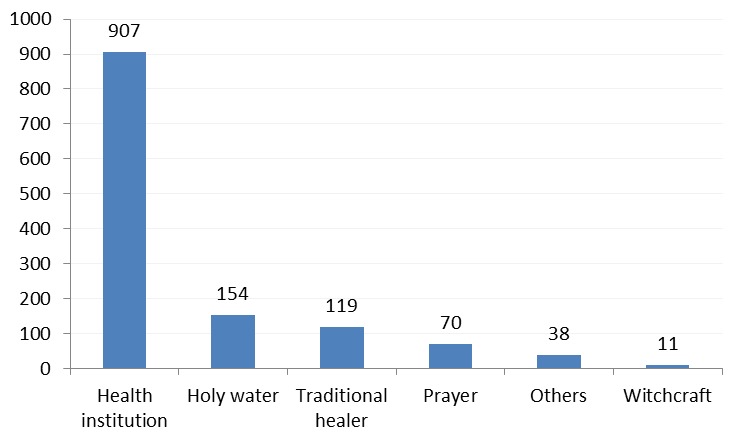
Distribution of sites AFP child first taken by families with acute flaccid paralysis child, Ethiopia, 2008

As indicated in Table 2 and Figure 2, a significant proportion of families 907(69.5%) went first to health institutions to seek medical care, while 398(30.5%) of parents took their child first to alternative traditional sites including holy water sites (11.8%), traditional healers (9.1%), and prayer places (5.4%). On the other hand over half of the families with AFP child reported practicing various home measures. Forty percent of the parents said that the child's father decided where to take the child first for medical care, while 29% were decided by the child's mother, and only in 23% of the cases, a decision was made by both parents. The majority of the families (85.2%) reported that they took their child with AFP to the nearest health facility to seek medical care, and 564(62.1%) families reported that they travelled to 10kms to reach to the nearest health facility, while 344(37.9%) traveled more than 10kms. The median distance traveled by families was found to be 5km ranging from less than 1km to 45 km. The median km is higher in Gambella (11.8km) and Somali (11.67km) regions (Table 2). Five hundred and sixty seven (49.3%) families reported that they went first to a health facility within five days of onset of paralysis, while 38.7% went to a health facility within 6-14 days of onset of paralysis. On the other hand, 700(59%) sought any help including traditional sites within 5 days, whereas 31.2% of the families went to any site of help within 6 to 14 days of onset of paralysis. Overall, 88% of families with an AFP child went to health institution within 14 days of onset of paralysis, while families seeking any kind of help including traditional sites were found to be 1070(90.2%)). 222(29.1%) of the families took their child to the health center, while 127(16.7%) and (15.4%) to health posts and hospital respectively. Eleven percent of families first went to church and holy water (7.1%) (Table 3).

**Table 2:** Characteristics of AFP cases with health seeking questionnaire and examined by MSOs: Ethiopia, 2004-2008

Traditional measures taken at home	Number	%
Prayer	206	15.8
Holy water	127	9.8
Traditional treatment	105	8.1
Massage	207	15.9
Nothing	636	48.9
Other	19	1.50
Total		
**Decision made by where to take the child first**	1299	100
By both parents	271	21.7
By father	512	41.0
By mother	385	30.8
By relatives	58	4.7
By OTHERS	23	1.8
Total		
**Child taken to the nearest health institution**	1141	100
Yes	1045	85.2
No	182	14.8
Total	1227	100
**Distance to the nearest health facility(Km)**		
<10km	658	71.9
11-20km.	172	18.8
21-30km.	45	4.9
31-40km.	11	1.2
>40km	29	3.2
Total	947	100
**Days child taken to health facility**		
≤5.	567	49.3
6-10.	382	33.2
11-14.	63	5.5
15-20.	69	6.0
21-25.	18	1.6
26-30.	25	2.2
31+	27	2.2
Total	1151	100

**Table 3:** Health seeking behavior of families by different characteristics Ethiopia 2004-2008

Days AFP child taken to any site of help		
≤5.	700	59.0
6-10.	312	26.3
11-14.	58	4.9
15-20.	58	4.9
21-25.	17	1.4
26-30.	19	1.6
31+	23	1.9
Total		
**Name of first site visited n=762**	1151	100
Health center	222	29.1
Health station/clinic/health posts	127	16.7
Hospital	117	15.4
Church	86	11.3
Holy water site	54	7.1
Private HF including drug vendor	41	5.4
Traditional healer	39	5.1
Home(Found at home)	22	2.9
Prayer	6	0.8
Others	48	6.3
Total		
**Information on PEI and AFP surveillance**	762	100
Yes	1112	85.5
NO	188	14.5
Total	1300	100
Sources of Health information on PEI & AFP*		
Health personnel	1123	85.1
Radio	338	25.6
Newsletter	21	1.6
TV	30	2.3
No source	55	4.2
Others	47	3.6
Total*	1614	100
**Median days to any site of help by region**	**To health facility**	**To any site of help**
Addis Ababa	5.0	4.0
Afar	5.0	4.5
Amhara	3.0	5.0
Benshangule. Gumuz	7.0	7.0
Dire Dawa	7.5	8.0
Gambella	11.5	4.0
Hareri	2.0	2.0
Oromia	6.0	7.0
SNNPR	4.0	5.0
Somali	7.0	7.5
Tigray	4.0	7.0
National	4.0	6.0

* One respondent may have more than one source of health information

Respondents were asked whether they had been exposed to any information regarding polio eradication and acute flaccid paralysis surveillance and site the source of information. Less than 5% of the respondents had no any source of information on polio eradication and AFP surveillance, while great majority (85%) mentioned health workers and radio (25.6%) as a main source of information, while newsletter and TV were the very least source of information mentioned (Table 3).

The influences of socio-Demographic characteristics of the study population on health seeking behavior of parents' first visit to health institutions were investigated using the standard bivariate methods (Table 4) shows that Occupation, income, traditional home measure practices, decision made by relatives and distance travelled more than 10kms from the health facility showed significant associations with health care-seeking behavior of families first to health facilities with acute

**Table 4:** Biavariate analysis of selected socio-Demographic characteristics and impact on health seeking behavior of families with acute flaccid paralysis child

Characteristics	First health seeking to health facilities		Odds ratio	95% CI	P- Value
	Yes	No			
**Education**					
No formal education	545	274	0.6680	0.504-0.88061	0.261230
educated	295	98			
**Occupation**			0.5332	0.4055-0.7011	0.000005
Farmers	539	297			
Others	312	90			
**Religion**					
Orthodox Christian	634	281	0.9741	0.7523-1.2612	0.85213
Others	271	117			
**Income ETB**					
<200	554	276	0.6940	0.5263-0.9150	0.00940
+200	269	93			
**Traditional Home measure**					
Do nothing	562	64	8.8662	6.5617-11.9689	0.00000
Home measure	326	329			
**Decision made**					
Both families	822	335	2.7853	1.7586-4.4114	0.00006
OTHERS	37	42			
**Distance traveled Km**					
<10km	473	179	1.6663	1.2286-2.2599	0.00096
+10km	157	99			

Flaccid paralysis child. However, as demonstrated in Table 5 better educational status, being civil servant and higher family income were associated with health care seeking behavior to modern health facilities first than traditional sites (P<0.05). Education and religion showed no significant association in the bivariate analysis. In the multivariate logistic regression analysis, we employed all socio-demographic variable considered in the bivariate analysis, education, income, religion and occupation were not demonstrating statistically significant association with health care seeking behavior of parents to modern health facilities first visit (p>0.05), while traditional home measures, the decision made by relatives or care takers and distance traveled more than 10kms by parents to the first place of choice were still demonstrating a significant association in multivariate logistic regression analysis (p<0.05). Religion, education, income and occupation remain insignificant in the logistic regression analysis, while , occupation and income which were significant in the bivariate analysis turned out to be insignificant (Table 5), while education and religion remain insignificant in both analysis.

**Table 5:** Logistic regression analysis of selected socio-Demographic characteristics impact on health seeking behavior of Families with acute flaccid paralysis child, Ethiopia

Category	Odds Ratio	95% C.I.		P-Value
Decision made by distant relatives	0.5595	0.3665	0.8540	0.0071
Distance more than 10 km	0.5962	0.4117	0.8634	0.0062
Education	1.0264	0.6764	1.5577	0.9024
Home measures taken	0.1202	0.0804	0.1797	0.0000
Income	1.0401	0.5615	1.9266	0.9006
Occupation	2.3435	0.9816	5.5949	0.0551
Religion	1.1766	0.8042	1.7213	0.4023

## Discussion

In this study, over 90% of the respondents were from 5 regions, which may be due to the high population contribution or better surveillance sensitivity that lead for detection of many AFP cases and care seeking questionnaire were filled. On the other hand, we found that 69.8% of the families are going first to health facilities when their child developed acute flaccid paralysis, while over 30% are looking for other traditional sites. This is very worrisome as the surveillance system in this country is mainly health facility based. However, 88% of the families going to a health facility within 14 days of onset of paralysis and can meet the minimal required surveillance performance indicator for certification assuming that the surveillance system at facility level is very sensitive enough to pick all cases going to health institutions. Nevertheless, this is unlikely that in many circumstances cases are missed because of lack of awareness or misconception of the case definition of AFP.

In general, health service utilization during illness is poor in many developing countries. A study conducted by Fantahun M. and Degu G in Amhara Region of Ethiopia indicated that among reported sicknesses and deaths, only 59% and 39.1% of them visited health facility respectively [13]. In addition, the DHS 2000 Ethiopia reveled that only 44% of the families utilized some type of health service for a sick child [14]. In this study, first health facility visit by parents with AFP child was found to be 69.5% and lower for pastoralists families compared to settled families, which may be due to the nature of mobility and absence of health facilities. In general mobile pastoralist population, health service utilization is lower than the settled population as evidenced by a study done by Double T and Haile Mariam D on determinants of conventional health service utilization among pastoralists in northeast Ethiopia [15].

In this study over 51% of the families does some sort of traditional interventions at home level before seeking any medical help which is one of the reason for late detection of cases and this has been statistically significant both in bivariate and logistic regression analysis. Massage and prayer were the most practiced traditional measures at home including holy water treatment. In the multi variate logistic regression, we observed a protective factor of home measures to first going to health facilities, which may indicate that families are doing different traditional measures and taking the child first to modern health facilities considered as a final resort when families tried all sort of interventions. Many families have little awareness of the possible causes of acute flaccid paralysis and the need to take the child immediately to health institutions, and over 39% of the respondents said that the community prefers to go to other traditional sites than the health facility which raised again a concern for the program. The need for strengthening the existing active case search and sensitization activities beyond the conventional health facility is paramount important in the surveillance system and target all possible sites where a family with AFP child might go for help such as traditional healer, holy water site, prayer place, church, drug vendor and others.

Although the result shows that the majorities (80%) of the respondents said they have information regarding AFP surveillance and polio eradication, we also observed that not a small percentage of families are seeking help first to none conventional sites. The majority of respondents cited health workers as a source of information, this may be due to the fact that parents may have this information during a health facility visit after child paralyzed and may not measure the actual information at the community level.

The study disclosed that 85% of the AFP cases have taken at least one dose of Oral Polio Vaccine (OPV) according to the family's history and over 78% of the cases have taken three or more doses of OPV. However, 4.8% of the children with flaccid paralysis had no history of vaccination. In general, in African region among AFP cases < 5years investigated in 2005 only 45% had received at least three doses of Oral Polio Vaccine [16].The high vaccination coverage in this study may be explained by the in-depth interview of families about vaccination status by the medical surveillance officer filling the health seeking behavior questionnaire.

About 33.6% of the respondents take their children to seek medical help first to a wide range of traditional sites (other than health institutions) for consultation to the affected child for different reasons. This includes, among others family's perception of disease as a supernatural cause, preference of the families to stay at home and doing traditional treatment or go to a traditional healer or praying sites which delay the health seeking intention of families to modern health institutions. Apart from the perception of families in regard to the disease in favor of traditional treatment, health service availability may have contributed to the low-level utilization of health facilities first. One of the reasons is the distance to the nearest health institution. In our study, we found that 29% of the families travel more than 10km to reach to the nearest health facility, while 78% of them live within 10 km radius from the health facility. DHS findings indicated that distance as the main problem in two to three women questioned [14]. In multivariate logistic regression, we didn't observe the role of religion, income, and occupation in the process of health seeking behavior of families with AFP child. In a study conducted in Bangladesh distance to hospitals were found to be a risk factor leading to a delay notification [17].

## Conclusion

In conclusion apart from modern health facilities families with an AFP child first seek health service in different traditional healing sites before going to modern health facilities. Targeting these sites in the surveillance network will greatly improve the NP-AFP rate and stool adequacy rate. In addition to traditional measures taken at home, decision by relatives, neighbors and distance traveled to the nearest health facility appears to be the main contributing factors to health seeking first in modern health facilities by families with an AFP child.

### What is known about this topic

- Traditional healers are most common in Ethiopia;
- Despite the increase in modern health facilities, traditional healing sites are most common and popular, and are not in the network of disease surveillance.

### What this study adds

- The study provides valuable information on factors that enable the improvement of the surveillance system;
- The study also provides information on the first sites visited by parents or care taker with an AFP child;
- The study also indicates the need to have sensitive and strong surveillance, including traditional healing sites.

## Competing interests

The authors declare no competing interests. The views expressed in the perspective articles are those of the authors alone and do not necessarily represent the views, decisions or policies of the institutions with which they are affiliated and the position of World Health Organization.
